# Anti‐arthritic effect of chicken embryo tissue hydrolyzate against adjuvant arthritis in rats (X‐ray microtomographic and histopathological analysis)

**DOI:** 10.1002/fsn3.2529

**Published:** 2021-08-18

**Authors:** Igor Rzhepakovsky, Shahida Anusha Siddiqui, Svetlana Avanesyan, Mehmet Benlidayi, Kunaal Dhingra, Alexander Dolgalev, Natella Enukashvily, Tilman Fritsch, Volker Heinz, Stanislav Kochergin, Andrey Nagdalian, Marina Sizonenko, Lyudmila Timchenko, Marko Vukovic, Sergey Piskov, Wolf‐Dieter Grimm

**Affiliations:** ^1^ Institute of Live Science North Caucasus Federal University Stavropol Russia; ^2^ Technical University of Munich Campus Straubing for Biotechnology and Sustainability Straubing Germany; ^3^ DIL e.V. German Institute of Food Technologies Quakenbrück Germany; ^4^ Faculty of Dentistry Department of Oral and Maxillofacial Surgery Cukurova University Sarıçam/Adana Turkey; ^5^ Division of Periodontics Centre for Dental Education and Research All India Institute of Medical Sciences New Delhi India; ^6^ Department of General Dentistry and Pediatric Dentistry Stavropol State Medical University Stavropol Russia; ^7^ Center for Innovation and Technology Transfer Stavropol State Medical University Stavropol Russian Federation; ^8^ Institute of Cytology Russian Academy of Sciences St. Petersburg Russia; ^9^ LTD InCome Stavropol Russian Federation; ^10^ Periodontology, School of Dental Medicine Faculty of Health Witten/Herdecke University Witten Germany

**Keywords:** adjuvant arthritis, antiarthritic effect, chicken embryo tissue, food‐derived bioactive peptides, histopathological analysis, hydrolyzate, in vitro and in vivo assays, X‐ray microtomography

## Abstract

Finding new, safe strategies to prevent and control rheumatoid arthritis is an urgent task. Bioactive peptides and peptide‐rich protein hydrolyzate represent a new trend in the development of functional foods and nutraceuticals. The resulting tissue hydrolyzate of the chicken embryo (CETH) has been evaluated for acute toxicity and tested against chronic arthritis induced by Freund's full adjuvant (modified Mycobacterium butyricum) in rats. The antiarthritic effect of CETH was studied on the 28th day of the experiment after 2 weeks of oral administration of CETH at doses of 60 and 120 mg/kg body weight. Arthritis was evaluated on the last day of the experiment on the injected animal paw using X‐ray computerized microtomography and histopathology analysis methods. The CETH effect was compared with the non‐steroidal anti‐inflammatory drug diclofenac sodium (5 mg/kg). Oral administration of CETH was accompanied by effective dose‐dependent correction of morphological changes caused by the adjuvant injection. CETH had relatively high recovery effects in terms of parameters for reducing inflammation, inhibition of osteolysis, reduction in the inflammatory reaction of periarticular tissues, and cartilage degeneration. This study presents for the first time that CETH may be a powerful potential nutraceutical agent or bioactive component in the treatment of rheumatoid arthritis.

## Graphical abstract

Finding new, safe strategies to prevent and control rheumatoid arthritis is an urgent task. Bioactive peptides and peptide‐rich protein hydrolyzate represent a new trend in the development of functional foods and nutraceuticals. This study presents for the first time that CETH may be a powerful potential nutraceutical agent or bioactive component in the treatment of rheumatoid arthritis.

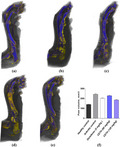

**Abbreviations**
BMD: Bone mineral density
BS/BV: Bone surface/bone volume
BV/TV: Percent bone volume
CETH: Chicken embryo tissue hydrolyzate
DMARD: Disease‐modifying antirheumatoid drugs
Micro‐CT: Computer microtomography
NSAID: Nonsteroidal antiinflammatory drugs
Tb.N: Trabecular number
Tb.Pf: Trabecular pattern factor
Tb.Sp: Trabecular separation
Tb.Th: Trabecular thickness

## 1 INTRODUCTION

Rheumatoid arthritis is the most frequent autoimmune disease and ranks first among inflammatory joint lesions (Firestein & McInnes, 2017). It is a severe chronic noninflammatory disease, which is characterized by inflammation accompanied by the destruction of joint structures, lesions of periarticular tissues, and bones (Kumar et al., 2016). Rheumatoid arthritis is characterized by a chronic course, steady progress, and a high prevalence of concomitant diseases, which significantly reduces the general functional status, quality of life, and is one of the main reasons for early disability (Myasoedova et al., 2019).

The etiopathogenesis of rheumatoid arthritis is very complex and has been under active study for many years (Scherer et al., 2020). Today, nonsteroidal antiinflammatory drugs (NSAID), disease‐modifying antirheumatoid drugs (DMARD), corticosteroids, and biological agents are the primary agents used to alleviate symptoms and slow the progression of rheumatoid arthritis (Law & Taylor, 2019; Van Vollenhoven, 2016). Regarding the cellular component of tissue engineering, NCSCs have been the focus of many efforts for cartilage regeneration. NCSCs are widely available from diverse tissues and are capable of expansion (self‐renewal) and multilineage differentiation into bone, cartilage, fat, muscle, and nerve (Arnold et al., 2010; Grimm et al., 2011, 2015; Keeve et al., 2013; Király et al., 2009; Kochkina et al., 2019; Widera et al., 2007 Jun; Zeuner et al., 2018).

However, the use of stem cell‐derived therapeutics, NSAID, DMARD, and corticosteroids has a fairly wide range of contraindications and can be accompanied by a range of adverse reactions, often limiting their clinical use (Dyadyk & Kugler, 2017; Oray et al., 2016). Therapy with biological drugs with high pharmacological selectivity and fewer side effects is very expensive, and not many patients can afford it (Lekander et al., 2012).

All this determines the importance of finding other effective, safe, and inexpensive strategies to combat rheumatoid arthritis. In autoimmune diseases like rheumatoid arthritis or systemic lupus erythematosus, the immune system turns against its own body and triggers inflammation. The International Society for Cellular and Gene Therapies (ISCT) and the International Society for Extracellular Vesicles (ISEV) recognize the potential of extracellular vesicles (EVs, including exosomes) from mesenchymal stromal cells (MSCs) and possibly other cell sources as treatments for OA (Grimm & Widera, 2019; Haque et al., 2019; Mellows et al., 2017; Skurikhin et al., 2019; Wang et al., 2017; Zeuner et al., 2016). The focus is shifting toward natural alternatives (Dudics et al., 2018; Grimm et al., 2016; Wang et al., 2019).

Perinatal tissues are of particular interest in this respect. The available literature increasingly mentions the antiarthritic effect of placental tissues (Park et al., 2012; Raines et al., 2017). Bioactive peptides and amino acids contained in placental tissues ensure their high biological and functional activity (Wang et al., 2018). Components of the placenta have expressed antiinflammatory and antioxidant properties (Heo et al., 2018; Kim et al., 2018) and possess antiapoptotic, antiosteoporotic, and regenerative effects (Bak et al., 2018; Pogozhykh et al., 2018). However, the use of placental tissues has economic, technological, epidemic, and ethical limitations. Therefore, in the scientific and industrial environment, there is no interest in finding alternative cheaper raw materials, more accessible, faster reproducible, and epidemiologically safe, but no less rich in biologically active compounds, especially protein and peptide nature. As such, a source of raw materials are now increasingly considered embryonic and extraembryonic tissues of birds, which are chemically not inferior to tissues of the placenta of humans and animals, and substances based on them in some countries are already used as effective nutraceuticals (Liu, 2007).

Hatching at different stages of a bird's egg is rich in peptides of different embryo tissues, including highly active peptides of polydirectional muscles with carnosine and anzerine (Kim et al., 2012), as well as functionally active amino acids of glutamate family, in particular, hydroxyproline (Rzhepakovsky et al., 2019).

Recent studies have shown that extracts of chicken embryonic and extraembryonic tissues have a pronounced antiinflammatory effect (Meram & Wu, 2017). Components of chicken embryonic tissues have high antioxidant and immunomodulatory properties (Li et al., 2012; Sun et al., 2014).

In previous studies using in vivo experiments, we have shown the antiinflammatory effect of biopreparations developed based on embryonic egg mass of birds (Areshidze et al.,^2015^, ^2018^)]. In in vitro experiments, we demonstrated antioxidant properties of peptide‐containing extracts of chicken embryonic tissues obtained by different hydrolysis methods (Rzhepakovsky et al., 2019).

However, despite the already confirmed biologically active properties of various substances based on chicken embryonic tissue, there is no information on their possible efficacy in rheumatoid arthritis in humans or laboratory animals. However, their component composition, especially the peptide–amino acid profile, has serious potential in this regard.

Therefore, the purpose of this study was to study the antiarthritic effect of chicken embryo tissue hydrolyzate (CETH) in adjuvant‐induced joint damage in rats, which is the closest possible model of rheumatoid arthritis in humans.

## 2 MATERIALS AND METHODS

### 2.1 Drugs and chemicals

Chemicals were obtained from the following sources: pepsin from porcine gastric mucosa (activity 600–1800 U/mg), hydrochloric acid 35% (Sigma‐Aldrich); pancreatin (activity): amylase 22,500 FIP E/g, lipase 22,500 FIP E/g, protease 1050 FIP E/g, (AppliChem); sodium nitrate ≥99.0%, potassium chloride ≥99.0%, 5‐sulfosalicylic acid dehydrate ≥99.0%, peptone from animal tissue, α‐cyano‐4‐hydroxycinnamic acid (for MALDI‐TOF MS), acetonitrile ≥99.0%, trifluoroacetic acid ≥99.0%, casein from bovine milk, trypsin from bovine pancreas ≥10,000 BAEE units/mg protein, formalin solution, neutral buffered 10%, isopropyl alcohol ≥99.7% (Sigma‐Aldrich); adjuvant complete Freund (Difco Laboratories); and diclofenac sodium (AMOLI ORGANICS Private Limited).

### 2.2 Materials

Certified fertilized chicken eggs were produced by white Leggorn breeds of Kumskaya poultry (Georgievsk, Russia) set. Cultivation of embryos up to 10 days of age was carried out under laboratory conditions in an incubator ILB‐0.5 (Russia) with automatic regulation of incubation parameters. During the incubation, the viability and level of embryos development were monitored using the PKYA‐10 ovoscope (Moscow, Russia). In accordance with the patent of the Russian Federation No. 2,560,845 (Timchenko et al., 2015), stimulation of embryos development was carried out using the AL‐01 Semicon (Moscow, Russia) medical semiconductor laser device. On the 10th day of incubation, eggs with developed embryos were placed for 7 days in a refrigerator at 2–6℃. The embryonic and extraembryonic tissues were then separated from the shell and ground using a Sterilmixer 12 knife homogenizer (PBI, Milan, Italy). The obtained substance was dried in a laboratory lyophilic dryer LS‐500 (Russia) and stored at 20℃ until use.

### 2.3 Preparation of chicken embryo tissue hydrolyzate (CETH)

The raw material was a sublimated embryonic egg mass from which the lipid fraction was removed by fivefold extraction with petroleum ether. It was stirred at 500 rpm on the magnetic stirrer (Heildoph, Germany), followed by drying of the defatted residue at 37℃ in an ES 20/60 thermoshaker (Biosan, Latvia). The resulting sublimate was carefully ground to a powdery state.

Five hundred mL of DW was mixed with 20 g of a protein‐containing powder, and putted into an ES 20/60 shaker thermostat for 30 min at 50℃. Then, 35% HCl was added to the solution to a concentration of 0.5% and held at 50℃ for 60 min with shaking 100 rpm in an ES 20/60 shaker thermostat. The resulting mass was then autoclaved at 125℃ for 60 min in the SPVA‐75‐1NN steam sterilizer. Porcine pepsin (0.1%) was added to a cooled substance at a concentration of 0.1% and the mixture was incubated in an ES 20/60 shaker thermostat for 120 min at 37℃.

The sample was then neutralized with 1 M NaOH to pH 7.0–7.3 (S400‐B pH meter) and pancreatin 2 mg/ml was added and incubated in a ES 20/60 shaker thermostat for 120 min at 37℃. Hydrolysis was stopped by boiling for 10 min. The resulting hydrolyzate was centrifuged (SL40R cooled centrifuge) at 4,700 rpm for 120 min at 2–4℃. The liquid obtained after centrifugation was subsequently filtered to remove enzymes and nonhydrolyzed proteins using a Vivaflow 50 filtration system with 0.2 μm and 30 kDa, and 10 kDa MWCO polyether sulfone membranes. Conductivity of hydrolyzate was 20.3 ± 2.2 mS/cm. Then, electrodialysis was carried out until conductivity hydrolyzate to 2.1 ± 0.1 mS/cm. The electrodialysis of the samples was carried out using the ED(R)‐Y/50 unit (MEGA a.s., Straz pod Ralskem, Czech Republic). The ED unit included three circulation streams, namely 300 ml of diluate (hydrolyzate), 300 ml of concentrate (main water, conductivity 0.43–0.48 mS/cm), and 300 ml of electrode solution (20 g/L NaNO_3_). The flow rates were 5 L/h for diluate and concentrate and 5 L/h for the electrode solution. The ED was conducted at an initial temperature of 22 ± 2℃. During the processing, the temperature of all streams did not rise above 30℃. After electrodialysis, the hydrolyzate was autoclaved at 120℃ for 10 min.

### 2.4 Physicochemical characterization of the CETH

#### 2.4.1 Hydrolysis degree

The degree of hydrolysis (DH, %) was determined by the amine nitrogen (AN)/total nitrogen (TN) ratio in the final hydrolyzate, where AN is the amine nitrogen content determined by the method of formaldehyde titration (Lahl & Braun, 1994) and TN is the content of total nitrogen determined titrimetrically by the Kjeldahl method.

#### 2.4.2 Dry matter

The amount of dry matter was determined by means of an Ohaus MB 25 (Ohaus Corporation, Parsippany, USA) moisture meter weighing (PRC) at 105℃.

#### 2.4.3 Ion analysis

The ionometry was carried out using a S400‐B pH meter (Mettler, Toledo, Spain).

#### 2.4.4 Protein and large peptide

Qualitative analysis for proteins and large peptides was carried out by reaction with sulfosalicylic acid.

#### 2.4.5 Peptides

Peptide concentration (PC) was determined using the biuret method (Rzhepakovsky et al., 2019) with 1% pepton as standard. Absorbance of the samples was measured at 540 nm in triplicates using UV spectrophotometer SF 102 (LLC “NPO Interfotofizika”, Moscow, Russia).

#### 2.4.6 Free amino acids composition

Analysis of free amino acid (FAA) composition was carried out with automatic amino acid analyzer Aracus (Aracus, Hennigsdorf, Germany).

#### 2.4.7 Matrix‐assisted laser desorption/ionization (MALDI) time‐of‐flight (TOF) mass spectrometry

Hydrolyzate, which demonstrated the most beneficial qualities from the point of view of further study, was subjected to proteomic analysis using MALDI‐TOF mass spectrometry. The hydrolyzate was centrifuged at 10,000 rpm for 4 min [MiniSpin microcentrifuge (Eppendorf AG, Hamburg, Germany)]. The supernatant (1 μl) was deposited on the MALDI plate. Pretreated and untreated samples were overlaid with 1 μl of matrix solution (saturated solution of a‐cyano‐4‐hydroxycinnamic acid in 50% acetonitrile and 2.5% trifluoroacetic acid). The matrix sample was cocrystallized by air drying at room temperature. Measurements were performed with a Microflex mass spectrometer (Bruker Daltonik) using Daltonics FlexControl software (version 3.3.64). Spectra were recorded in the positive linear mode (laser frequency, 60 Hz; ion source 1 voltage, 19.4 kV; ion source 2 voltage, 17.3 kV; lens voltage, 9.1 kV; and mass range, 0–20,000 Da). The internal calibration was performed using of the mass test standard MBT (Bruker Daltonics). For each spectrum, 4000 shots from different positions of the target spot (automatic mode) were collected and analyzed. Protein identification was performed using the BIOPEP database (Minkiewicz et al., 2008).

### 2.5 In vitro antiarthritic activity

#### 2.5.1 Inhibition of protein denaturation

The study was conducted according to the methodology presented in the work of Niazi et al. (2017). The reaction mixture (5 ml) consisted of 0.2 ml of fresh egg albumin, 2.8 ml of phosphate salt buffer (pH 6.3), plus 2.0 ml of different concentrations (3.75, 7.5, and 15.0 mg/ml) of CETH. The same volume of bidistilled water was controlled. The reaction mixtures were incubated at 37℃ for 15 min and then heated at 70℃ for 5 min. After cooling, the absorption was measured at 660 nm with UV spectrophotometer SF 102 (NPO INTERFOTOFIZIKA, Moscow, Russia). The percentage of protein denaturation inhibition was calculated using formula (1):

(1) $$Percentage\;of\;inhibition\;\left(\%\right)=\frac{Abs\;control‐\;Abs\;sample}{Abs\;control}\times 100$$

#### 2.5.2 Proteinase inhibitory action

The analysis was performed according to the methodology described by Chandra et al. (Chandra et al., 2015) with some modifications. The reaction mixture of 2.0 ml included 250 µl trypsin, 1.0 ml 25 mM Tris‐HCl buffer (pH 7.4), and 1.0 ml of different concentrations (3.75, 7.5, and 15.0 mg/ml) of CETH. The same volume of bidistilled water was controlled. The mixture was incubated at 37℃ for 5 min. 1.0 ml 0.8% (w/v) casein was added. The mixture was incubated for another 20 min. To stop the reaction, 2.0 ml of 70% (v/v) of chloric acid was added. Then, the turbid suspension was centrifuged. The optical density of supernatant at 280 nm was measured. The inhibition percentage was calculated by the above formula.

#### 2.5.3 Effect on membrane stabilization

The analysis was performed according to the method described by Demchenkov et al., (2021) and Shilpa et al. (2018). Fresh rat blood was collected in centrifuge tubes containing 200 mM 0.5 ml EDTA. The tubes were centrifuged at 3,000 rpm for 15 min and washed three times with an equal volume of physiological solution. The volume of RBC was measured and reduced as a 10% suspension with a physiological solution.

The reaction mixture (4.5 ml) included 2.0 ml of hypotonic physiological solution (0.25% NaCl), 1 ml of 0.15 M phosphate buffer (pH 7.4), and 1 ml of CETH (3.75, 7.5, and 15.0 mg/ml) in physiological solution. A similar volume of isotonic physiological solution was controlled. 0.5 ml of 10% rat RBC was added to the physiological solution. The mixtures were incubated at 56℃ for 30 min. Tubes were cooled under running tap water for 20 min. Mixtures were centrifuged at 3000 rpm for 10 min. The optical density of supernatants was measured at 560 nm. The percentage of stabilizing activity of the membrane was calculated by the formula (2):

(2) $$\%\;Membrane\;stabilization=\frac{Abs\;control‐\;Abs\;sample}{Abs\;control}\times 100$$

All studies of antiarthritic activity of CETH in vitro were conducted in a threefold repetition. Sodium diclofenac (200 µg/ml) was used as a comparison drug.

### 2.6 Animals

The experiment was conducted on male white rats of the Wistar line at the age of 10–12 weeks. The animals were kept in plastic cells in a laboratory vivarium under controlled environmental conditions (temperature 18–22℃, relative humidity 50%–65%, and 12 hr lighting cycle). Rats were kept on a standard food ration with free access to food and water. Before the experiment, the animals were acclimatized within 2 weeks.

All manipulations with animals were conducted in strict accordance with the Guide for the Care and Use of Laboratory Animals (National Research Council, 2011). The experiment was approved by the local bioethics committee of the Institute of Living Systems of North Caucasus Federal University (Study № 2020–003, Protocol № 4 of 22.09.2020). Every effort has been made to minimize animal suffering and reduce the number of rats used.

### 2.7 Acute oral toxicity

Toxicity assessment was performed on female white rats of Wistar line weighing 190–210 g according to OECD Test 425 recommendations (OECD, 2008). The test for limit values was carried out by successive use of five animals with an interval of 48 hr. CETH was administered to the animals after night fasting and weighing in a dose of 2000 mg/kg through a stomach tube. After CETH administration, the animals were monitored for any clinical manifestations of toxicity every hour during the first 4 hr and then every day for the next 2 weeks. Weight, feed, and water consumption were recorded daily. On the 15th day after euthanasia of animals, pathomorphological assessment of vital organs for any pathological changes was made.

### 2.8 Experimental protocol

After acclimatization within 14 days, the animals were randomly divided into five groups of six rats in each as follows:

Group I: Healthy control.
Group II: Adjuvant arthritis control.
Group III: Adjuvant arthritis rats with 5 mg/kg diclofenac sodium treatment.
Group IV: Adjuvant arthritis rats with 60 mg/kg CETH treatment.
Group V: Adjuvant arthritis rats with 120 mg/kg CETH treatment.

Arthritis was simulated by a single intradermal injection of 0.1 ml of full adjuvant Freund (modified Mycobacterium butyricum, CAS 9007–81–2, containing: 85% Drakeol 5NF, 15% Arlacel A, and 0.1% M.butyricum dry cells) in the pillow of the right hind leg of the rat. Animals were anesthetized by short‐term inhalation of ether during the injection because the viscous nature of the adjuvant makes it difficult to administer and painful (Bihani et al., 2014).

Manipulation was carried out in sterile conditions in the treatment room for animals far from the places where they were kept.

Animals of groups III, IV, and V received drug treatment for 14 consecutive days from 15 to 28 days after the adjuvant administration. CETH and diclofenac sodium was administered orally in the volume of 10 ml/kg of body weight once a day. The rats of groups I and II were given a carrier (water) in the same amount for 14 days. CETH antiarthritic activity was evaluated on injected animal paws.

### 2.9 Micro‐CT analysis

Microtomography of animal paws was performed ex vivo using a micro‐CT system (SkyScan 1,176; Bruker micro‐CT, Kontich, Belgium). For this purpose, the right hind legs were selected by cutting soft tissues and bone above the ankle joint during the autopsy.

Scanning protocol in Skyscan 1176 (10.0.0.0, Bruker‐microCT, Kontich, Belgium) for rat hind legs: 65 kV X‐ray source voltage acceleration, 380 µA X‐ray source current, Al 1 mm filter, 17.74 µm pixel size, 360º tomographic rotation, 0.3º shooting pitch, and four frames averaging.

Microtomographic images of bones and joints were reconstructed using Nrecon software (version 1.7.1.0, Bruker, Kontich, Belgium). Uniform positioning and selection of a certain area of the reconstructed object was performed in DataViewer (version: 1.5.6.2, Bruker, Kontich, Belgium).

The analysis of microtomographic bone data was carried out by means of CTAn software (version: 1.18.4.0, Bruker, Kontich, Belgium), visualization was carried out in CTvox software (3.3.0r1403, Bruker‐microCT; Nagdalian et al., 2021; Orhan, 2020; Sadyrin et al., 1889; Siddiqui & Ahmad, 2020). The following parameters were evaluated: trabecular mineral density (trabecular BMD, mg/cm3); BV/TV—percent bone volume; Tb. Pf—Trabecular pattern factor; Tb. Th—Trabecular thickness; Tb. Sp—Trabecular separation; BS/BV—Bone surface/bone volume; Tb.N—Trabecular number; and SMI—Structure model index.

Swelling volumes of feet and osteophytes were calculated using CTAn software (version: 1.18.4.0, Bruker, Kontich, Belgium), and visualization was performed in CTvox (3.3.0r1403, Bruker‐microCT, Belgium) and CTvol (2.3.2.0, Bruker‐microCT, Belgium). The results were documented with micro‐CT images in 2‐ and 3D images format.

The percentage of paw edema suppression was calculated from the average difference in paw volume in the experimental group treated and in the control group using the following formula (Siddiqui et al., 2021):

(3) $$\%\;inhibition=\frac{increase\;in\;paw\;edema\;\left(control\right)‐increase\;in\;paw\;edema\;\left(test\right)}{increase\;in\;paw\;edema\;(control)}\times 100$$

#### 2.9.1 Histological changes

After micro‐CT scanning, the animal paws were fixed in 10% buffered formalin solution for 72 hr. They were decalcified in a decalcifying solution of SoftiDec (Biovitrum, St. Petersburg, Russia) for 30 days. After that, they were dehydrated in isopropyl alcohol with subsequent soaking and entering into medical paraffin Histomix (Biovitrum, St. Petersburg, Russia). Histological slices with a thickness of 5–6 microns were made on sledge microtome MS‐2 (ATM‐practica, St. Petersburg, Russia). The ready slices were stained with hematoxylin and eosin, safranin O fast green, and Masson's trichrome followed by histopathological analysis.

Hematoxylin and eosin staining was used for general assessment of cell and tissue morphology and distribution. Safranin O fast green staining was used to determine the content of proteoglycans in the cartilage matrix (Schmitz et al., 2010). Masson's trichrome staining was used to observe changes in the morphology of bone trabeculae and the degree of unmineralized bone (Xu et al., 2016).

Evaluation of histological micropreparation was performed using laboratory microscope of research class Axio Imager 2 (A2) (Carl Zeiss Microscopy, Oberkochen, Germany) at ×50 magnification with image fixation with the help of specialized AxioCam MRc5 camera (Carl Zeiss Microscopy) and Zen 2 software (Carl Zeiss Microscopy).

A recently described comprehensive histological scoring system was used for histological evaluation of arthritis (Suranji Wijekoon et al., 2019). The ankle joints were characterized according to the following parameters:

- *Synovial hyperplasia:* score 0, absent; score 1, mild (5–10 layers); score 2, moderate (11–20 layers); and score 3, severe (20 layers).
- *Cellular infiltration [five high‐power magnification fields (HMF)]:* score 0, absent; score 1, mild (1%–10%); score 2, moderate (11%–50%); and score 3, severe (51%–100%).
- *Extension of pannus formation*: score 0, absent; score 1, mild; score 2, moderate; and score 3, severe.
- *Synovial fibrosis:* score 0, absent; score 1, mild (1%–10%); score 2, moderate (11%–50%); and score 3, severe (51%–100%).
- *Cartilage erosion:* score 0, absent; score 1, mild (1%–10%); score 2, moderate (11%–50%); and score 3, severe (51%–100%).
- *Cartilage degradation* (*Based on safranin O staining of proteoglycans):* score 0, none; score 1, mild loss (1%–10%); score 2, moderate loss (11%–50%); and score 3, severe loss (51%–100%).
- *Bone erosion:* score 0, none; score 1, minor erosion(s) observed only at HMF; score 2, moderate erosion(s) observed at low magnification; and score 3, severe transcortical erosions.

Scoring was executed blindly by two investigators and mean values were calculated.

#### 2.9.2 Immunohistochemistry analysis of caspase‐3 expression levels

The immunohistochemical study of caspase‐3 was conducted following the method described by Shafiey et al. (Shafiey et al., 2018) in some modification. Briefly, dewaxing and rehydration of ankle slices were performed, followed by buffer washing and heating in a water bath at 95℃ for 1 hr. Furthermore, to reduce the effect of endogenous peroxidase, the slices were placed in 0.3% H_2_O_2_ solution for 10 min. Then, the slices were incubated during the night at 4℃ with the primary antibody against caspase‐3 (Thermo Fisher Scientific, Fremont, USA). The cuts were washed with a buffer and incubated with the HRP secondary antibody Quanto polymer (Thermo Fisher Scientific) for 10 min at 27℃. After washing with distilled imaging water, a DAB solution was applied to the cuts (1 drop of DAB Quanto chromogen +1.0 ml of DAB Quanto substrate). After washing, the slices were dyed with hematoxylin, dehydrated in xylene, placed under cover glass, and studied with the Axio Imager 2 (A2) microscope (Carl Zeiss Microscopy).

The caspase‐3 expression level was quantitatively determined by densitometric analysis of digital images using Image J software (Bethesda, USA).

#### 2.9.3 Statistical analysis

The results were expressed as mean ± *SEM* (standard error mean). Statistical analyses were conducted with GraphPad Prism for Windows, Version 6.01 (GraphPad Software, San Diego, CA, USA). The statistical analysis was performed by one‐way variance analysis (ANOVA). A *p*‐value <0.05 was considered statistically significant.

## 3 RESULTS

### 3.1 Basic physicochemical parameters of CETH

The basic physiochemical parameters characterizing CETH are reported in Table 1.

**TABLE 1:** Basic physicochemical parameters of CETH, mean ±*SD* (*n* = 10)

The investigated parameter	CETH
Amount of dry matter, g/l	29.0 ± 0.9
Ionometry (pH)	7.03 ± 0.06
Total nitrogen (TN), %	0.4 ± 0.02
Amine nitrogen (AN), mg%	120.1 ± 7.5
Degree of hydrolysis (DH), %	30.0 ± 1.1
Number of peptides, mg%	1760.0 ± 98.0
Number of monosaccharides in recalculation for glucose, %	0.25 ± 0.02

### 3.2 Analysis of free amino acid

The content of amino acids and other hydrolysis products are reported in Table 2.

**TABLE 2:** Amount of amino acids and other hydrolysis products in the hydrolyzates, μg/ml, mean ±*SD* (*n* = 10)

Amino acids and other hydrolysis products	CETH
Aspartic acid (Asx^a^)	663.1 ± 18.1
*Threonine (Thr)	93.6 ± 3.6
Serine (Ser)	152.5 ± 4.5
Glutamic acid (Glx^b^)	387.3 ± 10.1
Glycine (Gly)	56.1 ± 1.1
Alanine (Ala)	125.1 ± 3.5
*Valine (Val)	113.1 ± 3.8
*Methionine (Met)	104.2 ± 4.2
*Isoleucine (Ile)	77.1 ± 2.7
*Leucine (Leu)	472.0 ± 19.6
Tyrosine (Tyr)	441.3 ± 17.3
*Phenylalanine (Phe)	529.0 ± 14.5
Histidine (His)	298.6 ± 8.1
*Tryptophan (Trp)	4.6 ± 0.4
*Lysine (Lys)	375.0 ± 11.2
Arginine (Arg)	530.3 ± 15.1
Proline (Pro)	30.5 ± 0.9
Phosphoserine (P‐Ser)	50.9 ± 1.6
Taurine (Tau)	37.2 ± 1.1
Urea	214.0 ± 6.9
Alpha‐aminoadipic acid (a‐AAA)	8.4 ± 0.6
Citrulline (Cit)	11.2 ± 0.9
Alpha‐aminobutyric acid (a‐ABA)	12.9 ± 0.9
Cystathionine (Cystha)	70.3 ± 2.7
H‐Cystine	35.1 ± 0.9
Gamma‐aminobutyric acid (g‐ABA)	16.9 ± 0.5
1Methylhistidine (1Mehis)	137.2 ± 4.1
Carnosine (Car)	568.5 ± 19.7
Anserine (Ans)	22.1 ± 0.9
Hydroxylysine (Hylys)	16.3 ± 0.5
Ornithine (Orn)	22.6 ± 0.7
Ammonia (NH3)	117.6 ± 3.3
Ethanolamine (EOHNH2)	31.6 ± 0.9
Hydroxyproline (Hypro)	1412.1 ± 39.1

^a^ Asx comprise Asp +Asn.

^b^ Glx comprise Glu+Gln.

* Essential amino acid.

CETH includes peptides, amino acids, and other hydrolysis products and monosaccharides. CETH contains significant amounts of amino acids such as aspartic acid, glutamic acid, leucine, tyrosine, phenylalanine, histidine, lysine, arginine, and hydroxyproline. Also, the analysis showed a high content of carnosine/anserine complex.

### 3.3 MALDI‐TOF mass spectrometry

**FIGURE 1:**
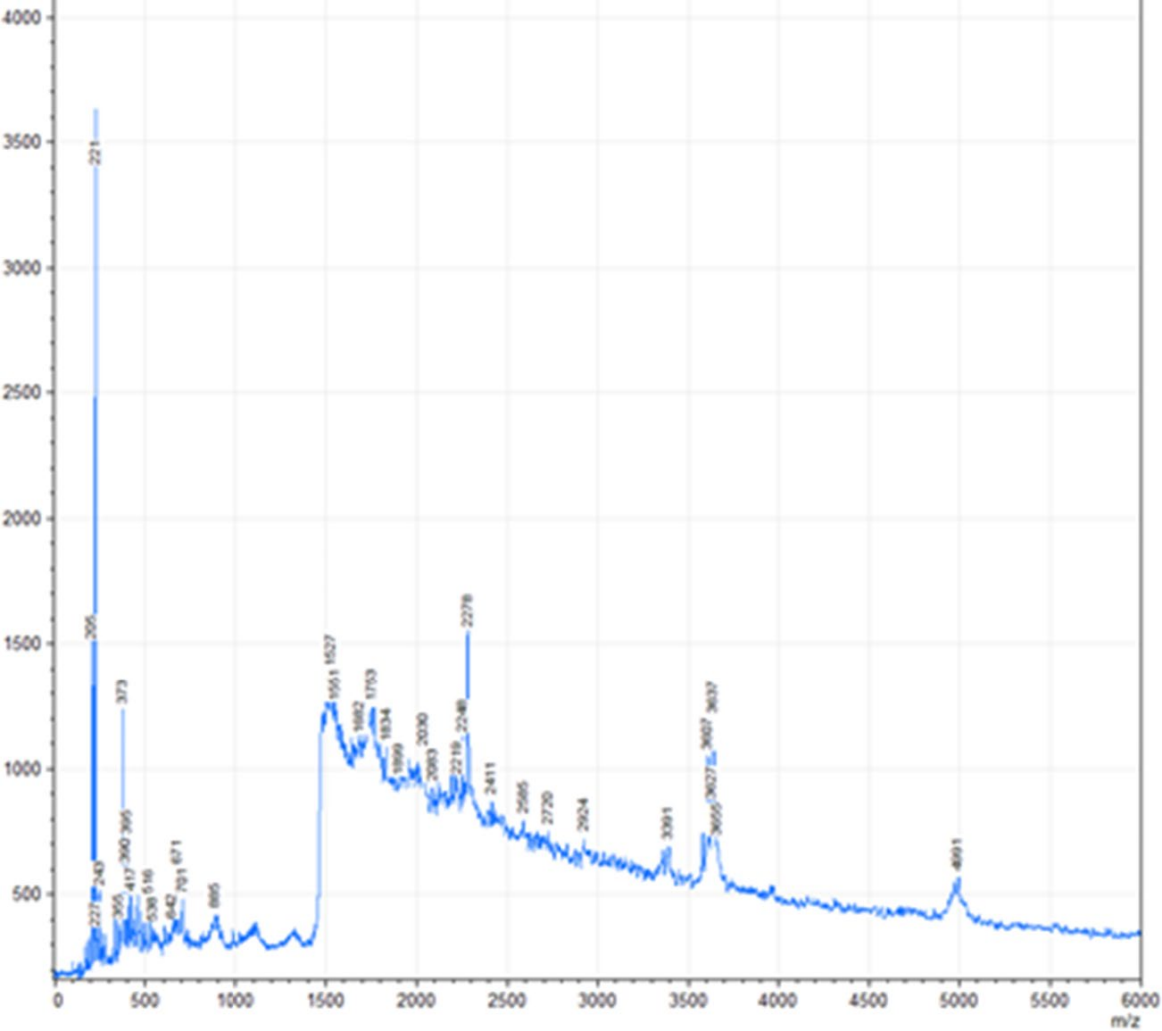
Data of the MALDI‐TOF mass spectrometry of CETH

The CETH study showed that the obtained mass spectra contain signals of different intensities in the range 200–5000 Da (Figure 1). There were about 15 signals with m/z up to 900 Da, about 20 signals in the 1500–3700 Da range, and one signal in the 4900–5000 Da range.

Analysis of the data obtained using the BIOPEP database showed that CETH contains peptides with predominantly antioxidant activity, dipeptidyl peptidase IV inhibitor activity, ACE inhibitor activity, and immunomodulatory activity, represented in the region up to 1800 Da (Table 3). Beyond this mass, there is no signal correspondence with biologically active peptides from the BIOPEP database.

**TABLE 3:** Characterization of the proteomic analysis of CETH (in accordance with the BIOPEP database)

Chemical mass, Da	ID	Sequence	Activity
243	3342	GPA	ACE inhibitor
	7810	KP	ACE inhibitor
	7837	PQ	ACE inhibitor
	8218	KP	Antioxidative
	8500	APG	Dipeptidyl peptidase IV inhibitor
	8519	KP	Dipeptidyl peptidase IV inhibitor
	8522	GPA	Dipeptidyl peptidase IV inhibitor
	8532	QP	Dipeptidyl peptidase IV inhibitor
	8858	PK	Dipeptidyl peptidase IV inhibitor
	8861	PQ	Dipeptidyl peptidase IV inhibitor
	9041	AGP	ACE inhibitor
355	8000	LHS	Antioxidative
373	7654	NKL	ACE inhibitor
390	3121	DGEA	Antithrombotic
	3341	FQP	ACE inhibitor
	8686	WW	Dipeptidyl peptidase IV inhibitor
395	8220	TFE	Antioxidative
417	8073	RWG	Antioxidative
	8600	WRG	Dipeptidyl peptidase IV inhibitor
456	3620	GRKP	Immunomodulating
516	8078	RWR	Antioxidative
	8604	WRR	Dipeptidyl peptidase IV inhibitor
538	7899	IYPF	Antioxidative
	8118	GALAAH	Antioxidative
	9015	AAPLAP	ACE inhibitor
642	3615	RNVRV	Immunostimulating
	8373	RHPHP	ACE inhibitor
	8469	RHPHP	Antioxidative
671	3367	GKKVLQ	ACE inhibitor
	9099	MTEEY	ACE inhibitor
	9100	MTEEY	Antioxidative
	9109	LIWKL	ACE inhibitor
701	8306	SVMPVVA	Antioxidative
885	9445	AIGVGAIER	Antioxidative
1527	3063	QPTIPFFDPQIPK	Immunomodulating
1753	9240	LVYPFPGPIPNSLPQN	ACE inhibitor

### 3.4 In vitro antiarthritic activity

The study of CETH in vitro antiarthritic activity included assessing inhibition of protein denaturation, the effect on membrane stabilization, and proteinase inhibitory activity. The study was performed for three different concentrations of CETH (3.75, 7.5, and 15.0 mg/ml) compared to a single dose of diclofenac sodium (200 mcg/ml). The results are presented in the table below (Table 4).

**TABLE 4:** Percent inhibition of different in vitro antiarthritic model of diclofenac sodium (200 mcg/ml), CETH (3.75 mg/ml), CETH (7.5 mg/ml), and CETH (15.0 mg/ml)

Treatment	Protein denaturation (%)	Membrane stabilization (%)	Proteinase inhibition (%)
Diclofenac sodium (200 mcg/ml)	66.36 ± 1.67^a^	58.42 ± 0.63^a^	91,37 ± 2,18^a^
CETH (3.75 mg/ml)	44.35 ± 1.12^b^	4.52 ± 0.11^b^	29.14 ± 0.73^b^
CETH (7.5 mg/ml)	66.70 ± 1.70^a^	30.47 ± 0.76^c^	77.0 ± 1.93^c^
CETH (15.0 mg/ml)	91.20 ± 2.28^c^	62.63 ± 1.57^a^	87.42 ± 2.24^a^

Note

Different superscript letters indicate statistically significant differences between the means (*p* <.05) for each parameter.

Protein denaturation is an etiopathogenesis‐proven mechanism of inflammation and development of rheumatoid arthritis. CETH showed high activity regarding protein denaturation inhibition. In the concentration of 7.5 mg/ml, CETH showed the same effect as diclofenac of sodium, and in the concentration of 15.0 mg/ml, it was significantly higher.

As erythrocyte membranes are similar to components of lysosomal membranes, inhibition of hypotonicity and lysis of erythrocyte membranes was taken as a measure of antiinflammatory activity mechanism.

CETH in doses 7.5 and 15.0 mg/ml effectively inhibited hemolysis induced by the hypotonic medium. In the concentration of 15.0 mg/ml, CETH showed membrane‐stabilizing activity on the same level as sodium diclofenac.

Proteinases are known to participate in the pathogenesis of arthritis. Leukocyte proteinases play an essential role in the development of tissue damage during inflammatory reactions. Accordingly, protection is provided by proteinase inhibitors (Oikonomopoulou et al., 2018). In this study, the inhibitory activity of trypsin was assessed since it is believed that it is trypsin that activates during the development of rheumatoid arthritis (OECD, 2008).

According to the results, the leading position in terms of the proteinase inhibitory effect was of sodium diclofenac. CETH (7.5 mg/ml) showed dose‐dependent activity, and in a concentration of 15.0 mg/ml, it was close to the comparison drug in terms of activity.

### 3.5 Acute oral toxicity

After oral administration of CETH at a dose of 2000 mg/kg of body weight, no visible signs of toxicity were observed in animals. No cases of mortality have been registered. Pathomorphological assessment of animals after 14 days of observation did not reveal any pathological changes.

Two doses of CETH 60 and 120 mg/kg of body weight were selected for further investigation ex vivo on white rats.

### 3.6 Micro‐CT analysis

The size of paw edema is one of the main criteria for assessing the antiarthritic activity of drugs in adjuvant‐induced arthritis. The volume of the paws was measured by micro‐CT and calculated using the CTAn software (version: 1.18.4.0, Bruker, Kontich, Belgium), visualization was performed in the CTvox software (3.3.0r1403, Bruker‐microCT, Belgium).

**FIGURE 2:**
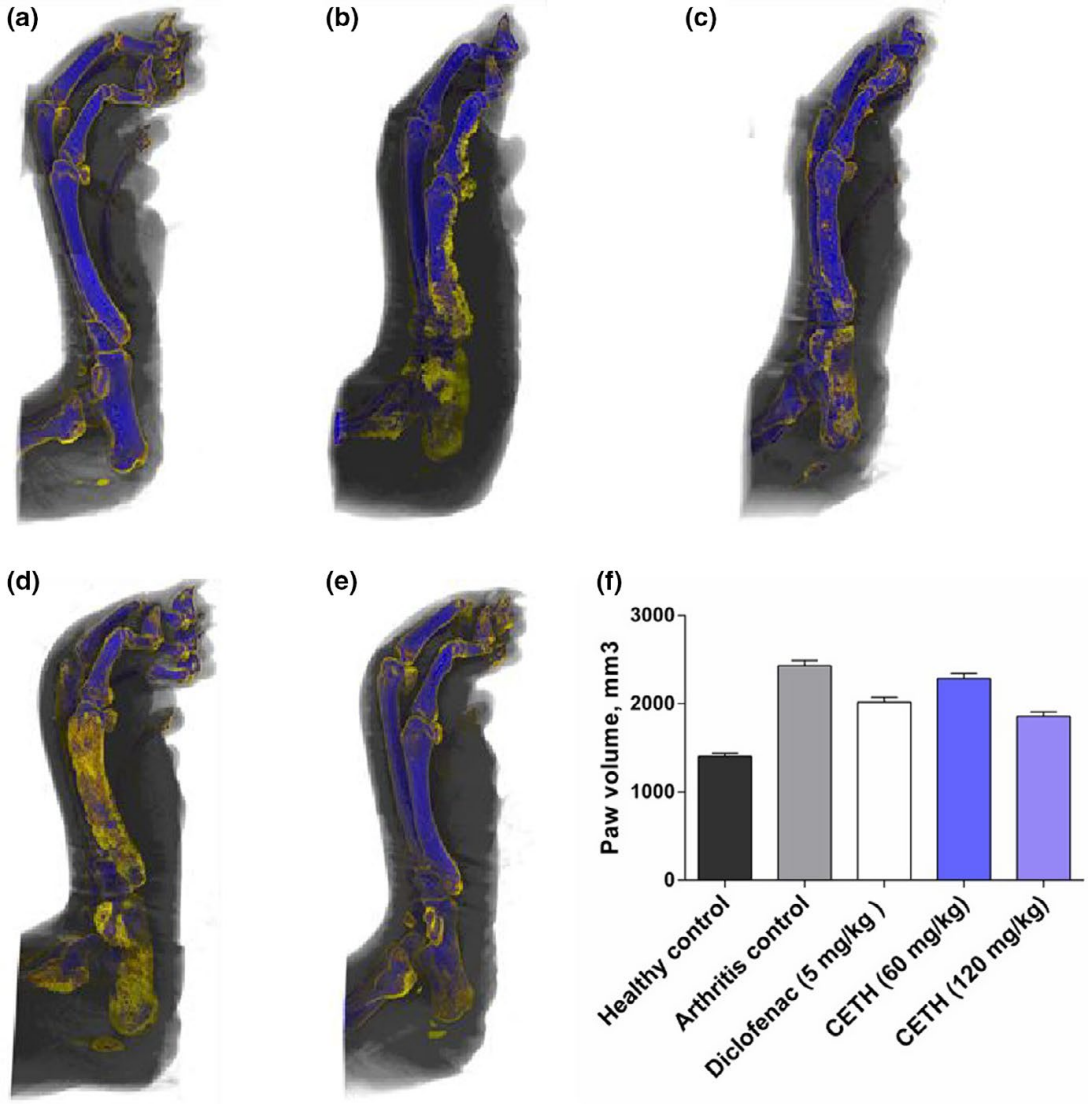
X‐ray microtomograms of representative hind paws of rats from five groups on day 28: (a) group I—healthy control; (b) group II—arthritis control; (c) group III—arthritis with diclofenac sodium (5 mg/kg) treatment; (d) group IV—arthritis with CETH (60 mg/kg) treatment; (e) group V—arthritis with CETH (120 mg/kg) treatment; and (f) injected paw volume (values represents shown as m±*SEM*)

In animals with adjuvant injection on the last 28th day of the experiment, pronounced peripheral edema of the injected paw was preserved (Figure 2b,f), which has been repeatedly described earlier in the work of other researchers (Almarestani et al., 2011).

As expected, the use of diclofenac demonstrated a noticeable therapeutic efficacy in adjuvant arthritis, in the form of an evident decrease in the swelling of the injected paw. The percentage of reduced inflammatory edema in group III was 21.1% (Figure 2c,f).

Representative X‐ray microtomograms on Figure 2d,e show that in comparison with the model group II, the use of CETH animals was also accompanied by a decrease in swelling. In group IV animals, the percentage of inhibition of inflammatory paw edema was 8.1%. The efficiency of CETH in the dose of 120 mg/kg was comparable to that of diclofenac and even slightly higher (23.5%).

Bone mass loss and remodeling caused by joint inflammation are the hallmarks of rheumatoid arthritis and are easily detected by measuring the mineral density of bone (BMD). The use of micro‐CT in this regard is a powerful tool and makes the BMD data a reliable biomarker with high sensitivity for the evaluation of antirheumatic drugs (Sevilla et al., 2015).

The value of trabecular BMD metaphyses of tibia obtained by micro‐CT in control group animals was 773.48 ± 19.32 mg/cm^3^. In rats of group II compared to healthy control, there was a loss of trabecular BMD by 16.2% (648.13 ± 15.80 mg/cm^3^). In animals receiving diclofenac and CETH in dose 60 mg/kg, BMD values exceeded those of group II and were 730.48 ± 18.72 and 752.65 ± 18.85 mg/cm^3^. The value of trabecular BMD in group V of animals after application of CETH in a dose of 120 mg/kg was as close to the control value as possible and was 772.24 ± 19.41 mg/cm^3^.

**FIGURE 3:**
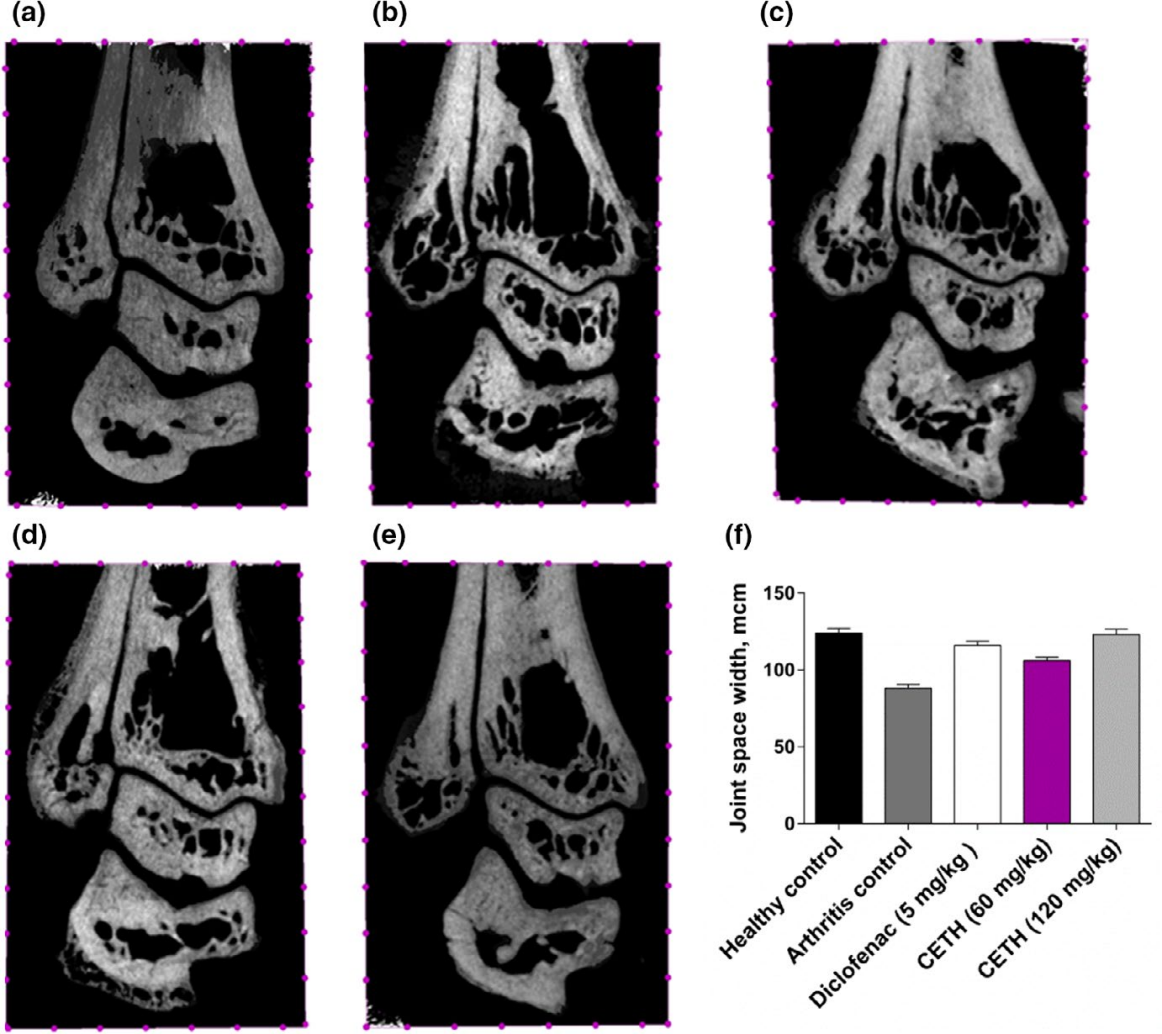
Micro‐CT image of the ankle joint of rats. Frontal slice: (a) group I—healthy control; (b) group II—arthritis control; (c) group III—arthritis with diclofenac sodium (5 mg/kg) treatment; (d) group IV—arthritis with CETH (60 mg/kg) treatment; (e) group V—arthritis with CETH (120 mg/kg) treatment; and (f) joint space width (values represents shown as m ± *SEM*)

The obtained micro‐CT images of the rat ankle joint area represented nondestructive visualization of changes that occurred. The frontal slices of the joints are shown in the picture. The bones forming the joint, namely the distal parts of the tibia and fibula, and the joint space are clearly shown (Figure 3).

To evaluate the therapeutic effect of CETH in simulated pathology, we focused on the width of the joint space, as there are data (Buckland‐Wright et al., 1995) that this value correlates with the thickness of cartilage. Changes in the joint space, expressed primarily in its narrowing, are an undeniable sign of rheumatoid arthritis. According to the results of Pfeil et al. (Pfeil et al., 2013), the X‐ray computer assessment is a useful criterion in monitoring the progression of arthritis and therapeutic assessment.

The micro‐CT images we received showed a significant narrowing of the joint space in Group II (29.0%) compared to the healthy control group (Figure 3a,b,f). Micro‐CTs of the ankle joint of animals receiving diclofenac, CETH (60 mg/kg), and CETH (120 mg/kg) show a marked increase in the width of joint space in comparison with the model group II by 31.8%, 20.5%, and 38.6%, respectively (Figure 3c–f).

**FIGURE 4:**
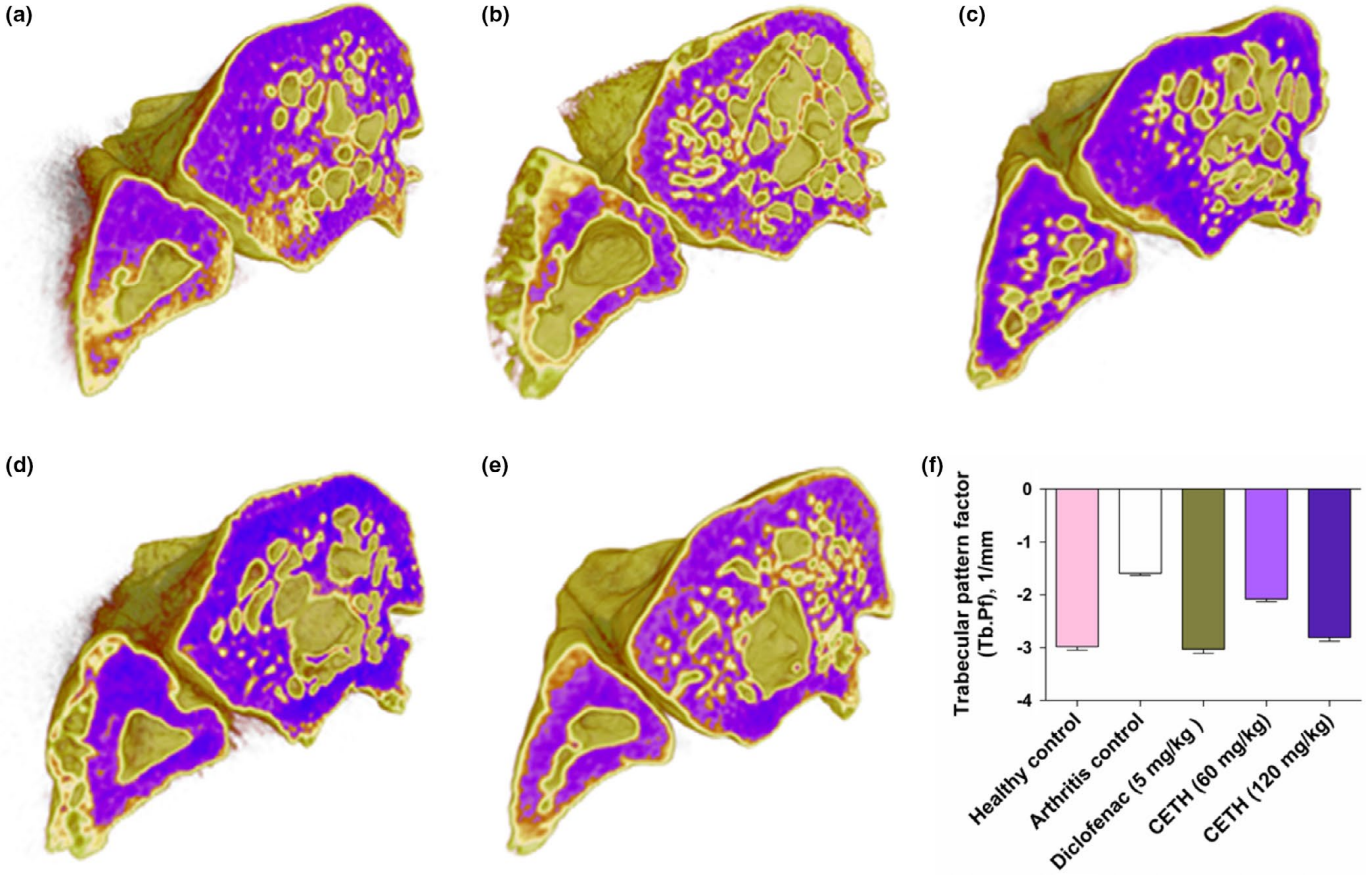
Periarticular bone resorption. Three‐dimensional reconstructions of micro‐CT images of the trabecular microarchitecture of the distal metaphysis of rat tibia. Transverse slice (a) group I—healthy control; (b) group II—arthritis control; (c) group III—arthritis with diclofenac sodium (5 mg/kg) treatment; (d) group IV—arthritis with CETH (60 mg/kg) treatment; (e) group V—arthritis with CETH (120 mg/kg) treatment; and (f) trabecular pattern factor (Tb. Pf), 1/mm (values represents shown as m ± *SEM*)

The introduction of adjuvant in the modeling of arthritis caused a marked loss of bone tissue and weakening of trabecular microarchitecture. In comparison with the group with simulated arthritis, the fragmentation of trabecular bone was detected in varying degrees (Figure 4).

According to 3D images in Group II, the tibia distal metaphysis was characterized by high porosity and loss of trabecular connections. A large proportion of bone trabeculars lost their normal architecture and looked like separate bone areas separated by extended spaces. This is consistent with the results of several researchers (Almarestani et al., 2011) and, according to Noguchi et al. (2008), can be caused by activation of inflammation mediators, by means of which stimulation of osteoclastic formation takes place, which eventually leads to periarticular bone resorption (Kotova et al., 2020).

Micro‐CT images of the distal metaphysis of the tibia in rats of group III treated with diclofenac (Figure 4c) and groups IV and V treated with CETH (Figure 4d,e) show the presence of a protective effect in relation to bone resorption. Group V animals had the largest effect. They still had areas of resorption, but the overall integrity of the trabecular architecture of the bone components of the joint, in particular, the tibia, was as close to the group I as possible. This was quantitatively confirmed by reliable differences in the value of the trabecular pattern factor (Tb. Pf) (Figure 4f), reflecting the connectivity of bone structures and negatively correlating with bone strength (Ito, 2007).

The average value of TBPf in group II almost twice exceeded this value in control group I. In groups III, IV, and V, a significant decrease in TBPf was observed in comparison with II. In sample III, which received diclofenac, TBPf decreased by 90.5%. In groups IV and V, TBPf decreased by 30.8% and 76.7%, respectively, compared to CETH.

This and other quantitative parameters of the trabecular microarchitecture of subchondral bone, reproduced by three‐dimensional microtomography analysis, are presented in Table 5.

**TABLE 5:** Microarchitecture parameters of tibia distal metaphysis by micro‐CT analysis (*M* ± m)

Groups	^1^BV/TV, %	^2^Tb. Pf, 1/mm	^3^Tb. Th, mm	^4^Tb. Sp, mm	^5^BS/BV, 1/mm	^6^Tb.*N*, 1/mm	^7^SMI
Healthy control	66.89 ± 0.45^a^	−2.98 ± 0.07^a^	0.31 ± 0.005 ^a^	0.50 ± 0.028^a^	9.14 ± 0.23^a^	2.29 ± 0.042^a^	−1.70 ± 0.12^a^
Arthritic control	52.98 ± 0.68^b^	−1.59 ± 0.04^b^	0.24 ± 0.003 ^b^	0.67 ± 0.036^b^	13.48 ± 0.34^b^	2.22 ± 0.050^a^	−1.08 ± 0.19^b^
Diclofenac (5 mg/kg)	55.46 ± 0.96^b^	−3.03 ± 0.08^a^	0.24 ± 0.005 ^b^	0.49 ± 0.031^a^	11.96 ± 0.41^c^	2.44 ± 0.093^a^	−1.34 ± 0.15^a^
CETH (60 mg/kg)	62.78 ± 1.86^a^	−2.08 ± 0.05^c^	0.28 ± 0.004 ^c^	0.55 ± 0.027^a^	12.06 ± 0.29^c^	2.35 ± 0.049^a^	−1.66 ± 0.27^a^
CETH (120 mg/kg)	69.90 ± 1.41^a^	−2.81 ± 0.07^a^	0.27 ± 0.007 ^c^	0.52 ± 0.045^a^	11.27 ± 0.31^c^	2.58 ± 0.073^b^	−1.75 ± 0.18^a^

Note

Different superscript letters indicate statistically significant differences between the means (*p* <.05) for each parameter. ^1^Percent bone volume; ^2^Trabecular pattern factor; ^3^Trabecular thickness; ^4^Trabecular separation; ^5^Bone surface/bone volume; ^6^Trabecular number; ^7^Structure model index.

The BV/TV ratio as a predictor of bone strength in group II was 20.8% lower than in group I. Groups IV and V that received CETH were characterized by 18.5% and 31.9% increase in BV/TV compared to group II, respectively. The use of diclofenac in group III did not statistically affect the value of BV/TV.

The values of Tb.N parameter in contrast to Kim & Kang's data (Kim & Kang, 2015) did not undergo statistically significant changes after the modeling of arthritis. This is probably due to the fact that the main bone loss in the periarticular zones was not due to the perforation of bone trabeculae, as is often observed in osteoporosis (Hayatullina et al., 2012), but due to the thinning of trabeculae. This is confirmed by the changes in Tb. Th. values we have detected.

The data on group distribution of the parameter Tb. Th, which is one of the criteria for osteoanabolic action, were presented interestingly. Group II was characterized by a reliable decrease in Tb. Th value in comparison with the control. The application of diclofenac in group III did not affect Tb. Th values, and it remained as low as in group II. At the same time, groups IV and V after the application of CETH showed a significant increase in the Tb. Th value in comparison with group II but remained low in relation to the Tb. Th values of group I of healthy controls.

The value of SMI, reflecting the loss of bone strength, in the model group II, increased by 36.5% compared to the control. Against the background of diclofenac application in group III, this index reliably decreased by 24.1%. The downward changes in SMI compared to the model group II in samples IV and V were 53.7% and 62.0%, respectively.

Group II was characterized by relatively high values of Tb. Sp. and BS/BV. These changes are another confirmation of bone mass loss, and according to Kim & Kang (2015), a quantitative sign indicating the formation of osteophytes.

**FIGURE 5:**
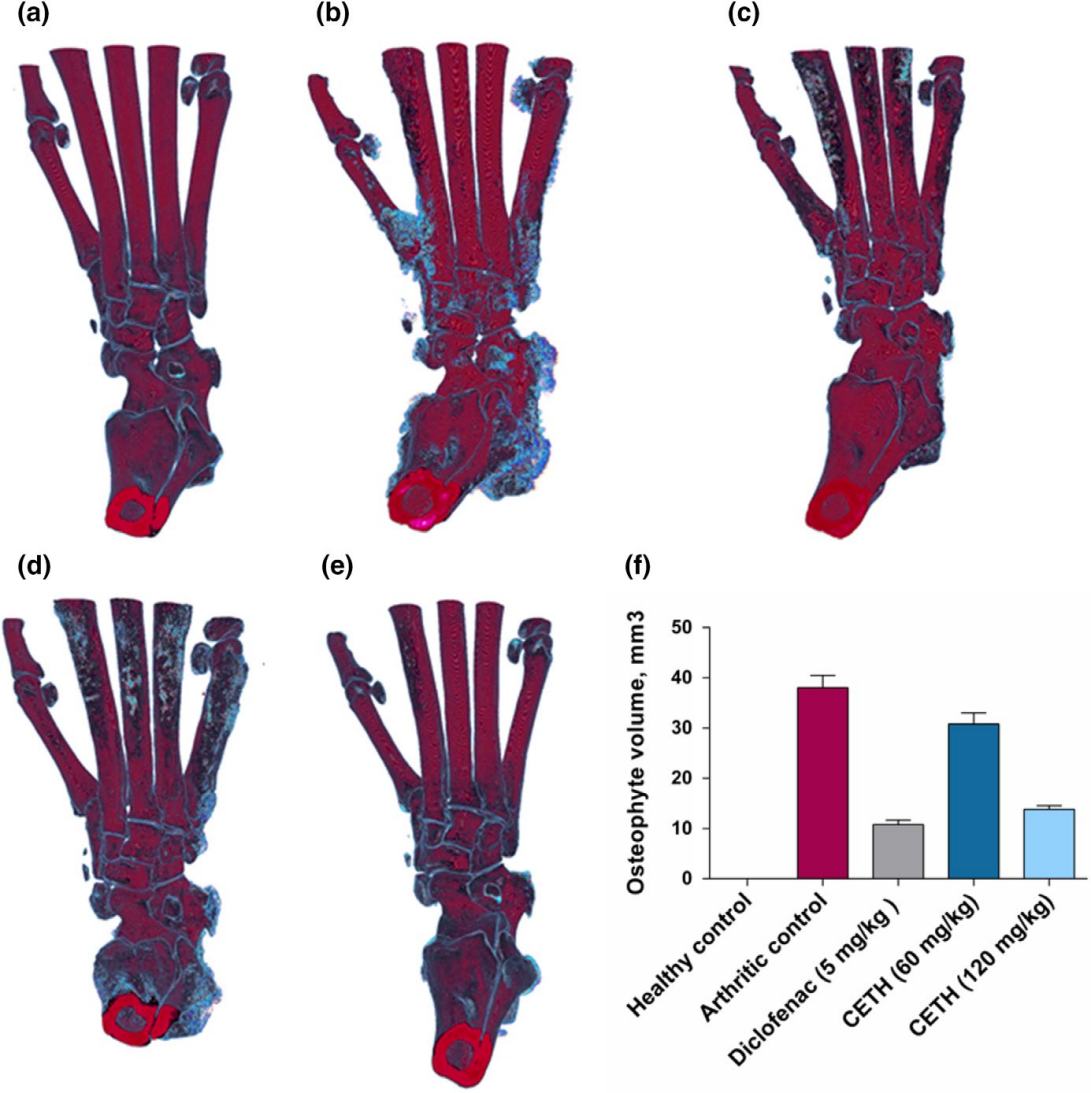
Visualization of osteophytes on three‐dimensional micro‐CT images of rat paw skeleton. (a) Group I—healthy control; (b) group II—arthritis control; (c) group III—arthritis with diclofenac sodium (5 mg/kg) treatment; (d) group IV—arthritis with CETH (60 mg/kg) treatment; (e) group V—arthritis with CETH (120 mg/kg) treatment; and (f) osteophyte volume, mm^3^ (values represents shown as m ± *SEM*)

In this study, following the study provided by Kim & Kang, (2015), osteophytes were visualized using CTvox and CTvol software (Bruker‐microCT) (Figure 5). In 3D images, they are represented as blue‐colored outgrowths with an uneven surface formed along the edges of joints and bones.

According to micro‐CT data, all groups of animals with induced arthritis were accompanied by osteophytosis. These pathomorphological changes are typical not only for osteoarthritis but also often occur in the development of adjuvant arthritis, which has been repeatedly confirmed in the works of other researchers (Almarestani et al., 2011; Wu et al., 2002).

The quantitative volumetric characteristics of osteophytes obtained differed greatly among the groups of animals studied. Osteophytes were not registered in animals of control group I (Figure 5a). The maximum volume of osteophytes formed was characterized by group II (Figure 5b). Oral administration of diclofenac (5 mg/kg) and CETH (60 and 120 mg/kg) to rats significantly prevented osteophytes development (Figure 5c,d,e). The effectiveness of CETH in a dose of 120 mg/kg was comparable to that of diclofenac (Figure 5f). This is confirmed by the above values of BS/BV and Tb. Sp, which in groups III, IV, and V were significantly lower than in the model group II.

### 3.7 Histopathological analysis

Since the possibilities of micro‐CT analysis to study cartilage structures are somewhat limited, histological analysis of the joint tissues of the white rat ankle was performed to evaluate the effectiveness of CETH antiarthritic action.

According to the results of the histological study, no inflammation or tissue destruction was observed in group I. In groups II, III, IV, and V, pathomorphological signs characteristic for adjuvant arthritis of various degrees of severity were found (Table 6).

**TABLE 6:** Histological score of rats

Groups	Synovial hyperplasia (0–3)	Cellular infiltration (0–3)	Pannus formation (0–3)	Cartilage erosion (0–3)	Bone erosions (0–3)
Arthritic control	2.0 ± 0.24	2.3 ± 0.24	2.0 ± 0.40	2.0 ± 0.31	2.4 ± 0.40
Diclofenac (5 mg/kg)	0.6 ± 0.33*	1.3 ± 0.3*	0.16 ± 0.16*	0.6 ± 0.33*	0.83 ± 0.33*
CETH (60 mg/kg)	1.2 ± 0.30	1.3 ± 0.30*	0.83 ± 0.33*	1.2 ± 0.30	2.2 ± 0.31
CETH (120 mg/kg)	0.6 ± 0.33*	0.6 ± 0.33*	0.16 ± 0.16*	0.6 ± 0.33*	0.6 ± 0.33*

* *p* <.05 versus arthritic control.

**FIGURE 6:**
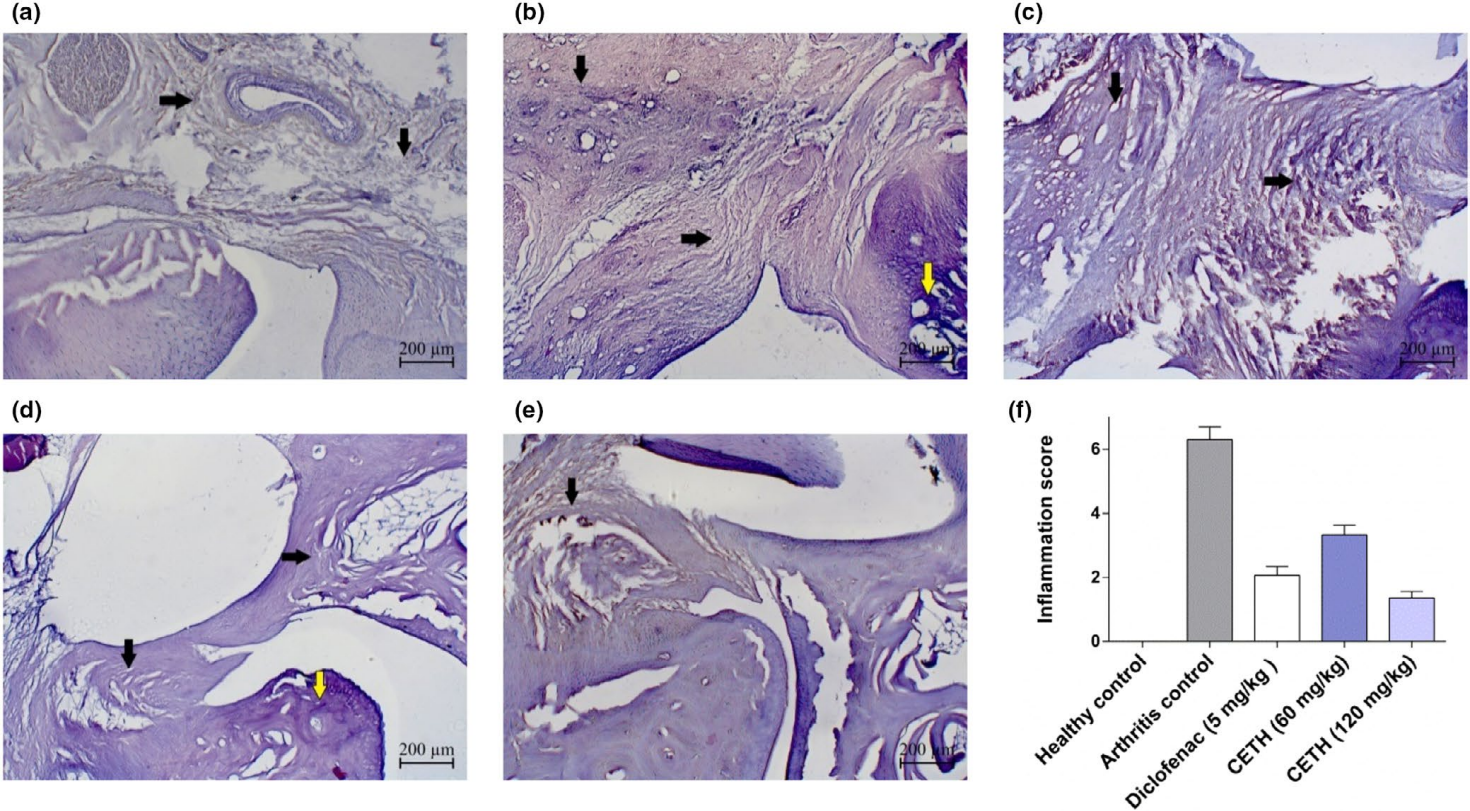
State of periarticular tissues. Coloring with hematoxylin and eosin, ×50: (a) group I—healthy control. Periarticular tissues are normal (indicated by arrows); (b) group II—arthritis control. Significant thickening and signs of marked inflammatory reaction in periarticular tissues (indicated by arrows), osteophyte (yellow arrow); (c) group III—arthritis with diclofenac sodium (5 mg/kg) treatment. Mild infiltration with moderate swelling in periarticular tissues (indicated by the arrows); (d) group IV—arthritis with CETH (60 mg/kg) treatment. Mild periarticular infiltration with mild swelling (indicated by the arrows), slight osteophyte (yellow arrow); (e) group V—arthritis with CETH (120 mg/kg) treatment. Mild periarticular tissue infiltration (indicated by the arrow); and (f) general joint inflammation expression (values shown as m ± *SEM*)

In the ankle joint of animals of group II, hyperplasia of cells of synovial shell and its thickening and the presence of dark bulbous villi on synovial intima were observed. Significant signs of inflammatory reaction in periarticular tissues with increased vascularization, swelling, infiltration of inflammatory cells, and pannus formation were registered (Figure 6b).

In all experimental groups with the use of drugs, the inflammatory response in periarticular tissues was characterized by a much lower level than in animals without treatment. In groups using diclofenac and peptides in the dose of 60 mg/kg, the manifestations ranged from mild to moderate infiltration by inflammatory cells with moderate swelling (Figure 6c,d). The lowest level of general joint inflammation was characterized by animals of group V receiving CETH 120 mg/kg (Figure 6e).

**FIGURE 7:**
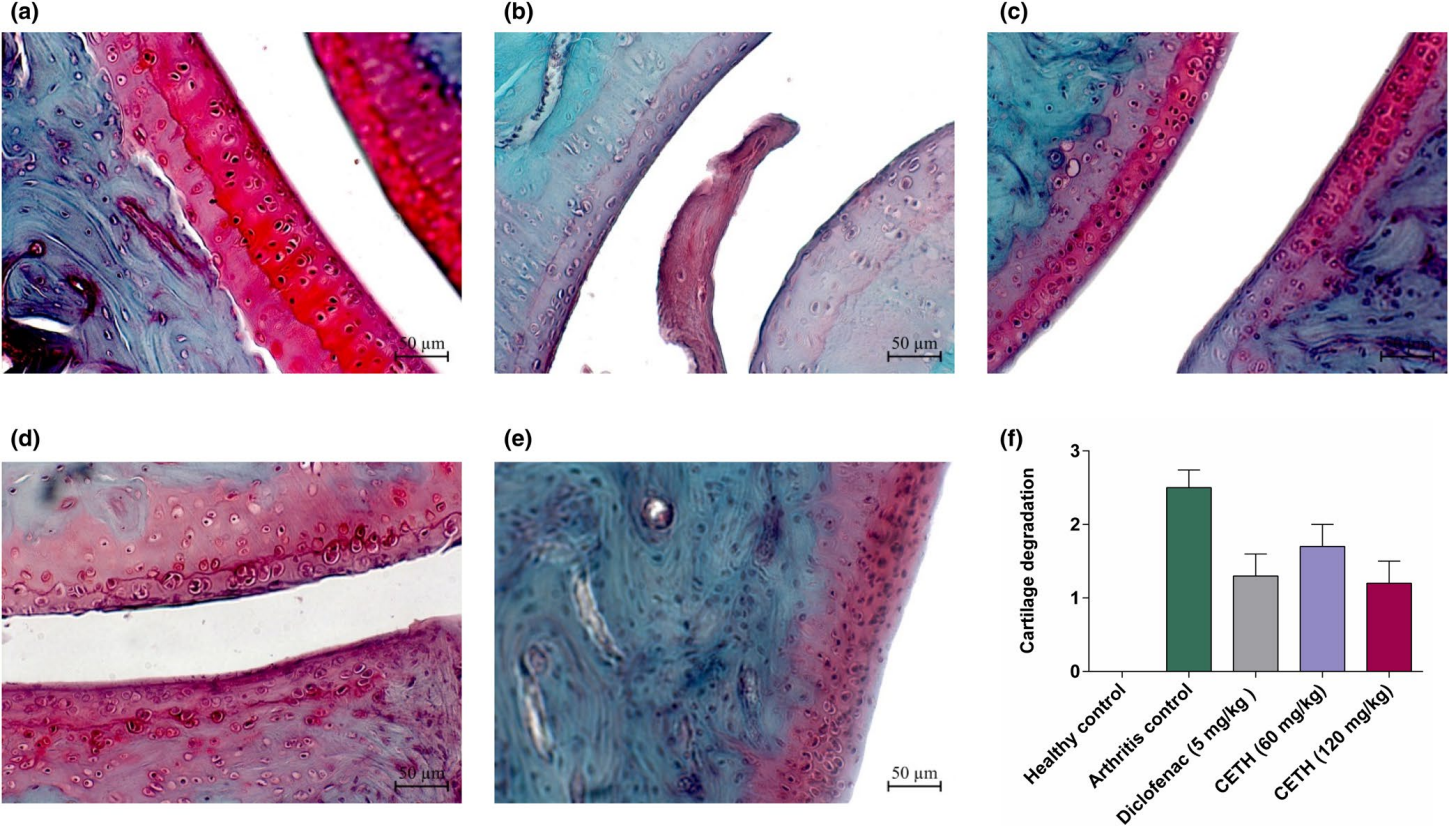
The condition of cartilage tissue of the joint. Coloring with safranin O fast green, ×200: (a) group —healthy control. Joint surfaces and chondrocytes are normal (intense uniform color (red) safranin O); (b) group II—arthritis control. Joint surfaces with signs of dystrophic and necrotic chondrocyte lesions. Severe protein depletion, which is indicated with the color (red) intensity of safranin O stain; (c) group III—arthritis with diclofenac sodium (5 mg/kg) treatment. Joint surfaces with irregularities, and irregular content of proteoglycans; (d) group IV—arthritis with CETH (60 mg/kg) treatment. Joint surfaces with irregularities and curved groups of two cells. Foci of proteoglycans with intense staining (red) safranin O; (e) group V—arthritis with CETH (120 mg/kg) treatment. Joint surfaces with irregularities and small cracks. Proteoglycans are retained in sufficient amount (uniform, but less marked than normal staining (red) safranin O); (f) cartilage degradation (based on safranin O staining of proteoglycans) (values represented as m ± *SEM*)

The degree of cartilage degeneration in group II was the most pronounced and was characterized by swelling and vacuumization of the matrix, and the development of dystrophic and necrotic lesions of chondrocytes. Surface erosions and splits were found in cartilage tissue as well as cartilage loss of proteoglycan (proportional to safranin O staining; Figure 7). In severe cases, there was thinning and impoverishment of cartilage tissue cells, with proliferation foci, there were found out curved groups of chondrocytes of 2, 3, and more cells.

Histological evaluation of the degree of cartilage tissue degeneration in the region of ankle joints in animals of groups III and V indicates small irregularities, erosions, and some animals have cracks. In the groups of animals, which used diclofenac 5 mg/kg and CETH 120 mg/kg, there were practically no changes on the part of chondrocytes. There were also no significant differences in the semiquantitative calculation of cartilage damage, through the accounting of proteoglycan accumulation in cartilage (1.3 ± 0.3 points and 1.2 ± 0.33 points, respectively). When using CETH 60 mg/kg in animals, IV showed more pronounced histological changes in the joints, which were characterized not only by irregularities and erosions but also by the presence of corrugated groups of chondrocytes with two cells in a group. The degree of cartilage degeneration by safranin O staining in this group was 1.7 ± 0.3 points.

**FIGURE 8:**
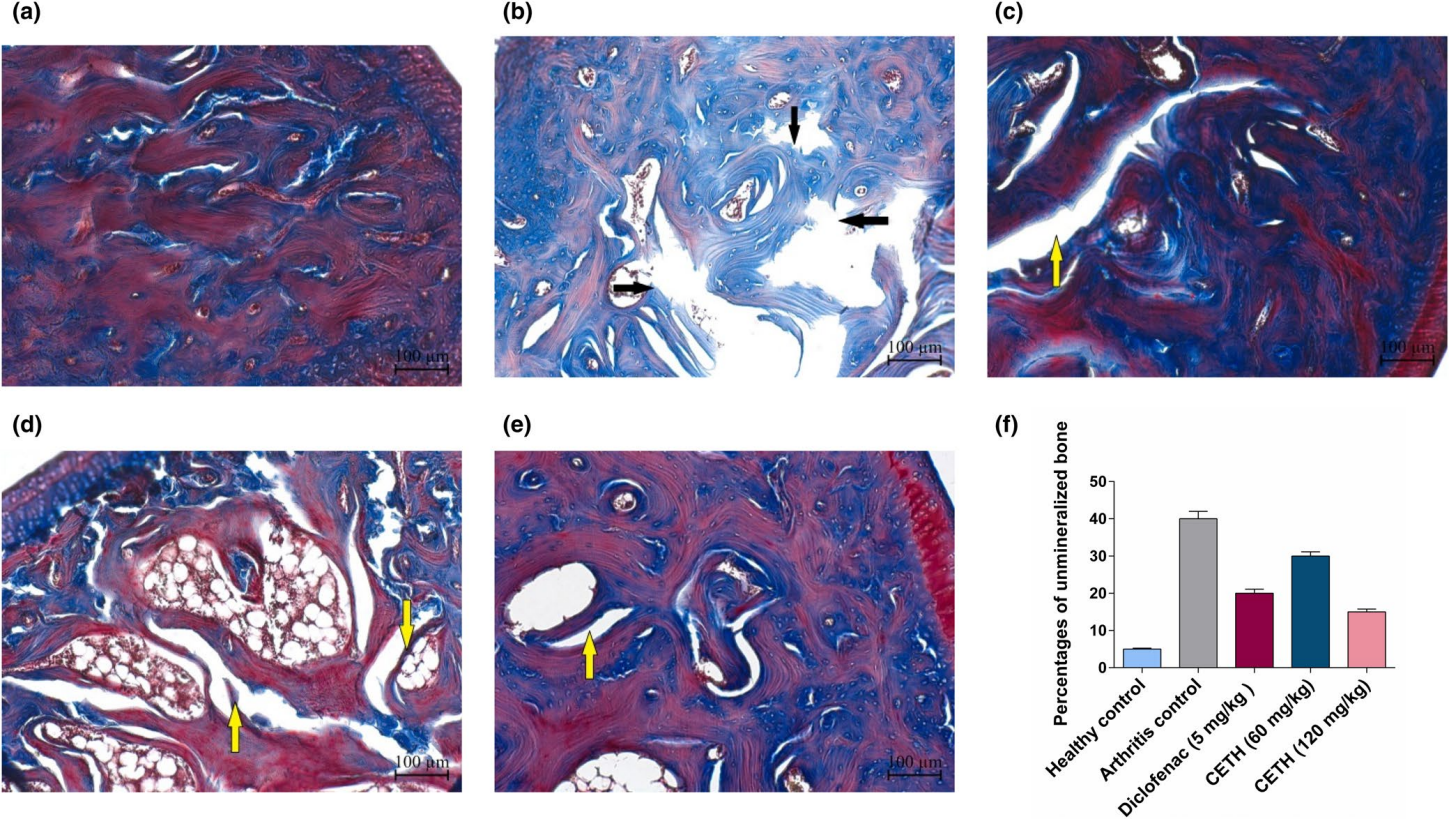
**State of the bone tissue of the rat pelvic bone** coloring with Masson's trichrome, ×50: (a) group I—healthy control. Normal pelvic bone; (b) group II—arthritis control. Extensive areas of bone thinnings, fragmentation, and elimination, pronounced osteoclastic activity of the tuber bone (osteolysis) (indicated by arrows); (c) group III—arthritis with diclofenac sodium (5 mg/kg) treatment. Local area of pelvic bone destruction (indicated by arrow); (d) group IV—arthritis with CETH (60 mg/kg) treatment. Several localized areas of separation and fragmentation of the pelvic bone (indicated by arrows); (e) group V—arthritis with CETH (120 mg/kg) treatment. Nonsignificant areas of pelvic bone destruction (indicated by arrow); (f) percentages of unmineralized bone (values represents shown as m ± *SEM*)

In all animals of group II, during the histological analysis of an ankle joint, pronounced changes in bone tissue were found; in particular, trabecular osteolysis. Irregular and disordered bone structures were observed, with a low level of mineralization (by Masson trichrome staining). Mineralized old trabecular bone and unmineralized new bone were stained red and blue, respectively, by Masson trichrome staining (Figure 8).

In groups III, IV, and V, histological analysis of bone tissue changes showed less pronounced osteolysis. These groups were characterized by a much lower degree of resorption of the medullary tibia region. In ankle bone, the weak level of trabecula resorption was visualized in the animals of group IV, which received CETH 60 mg/kg, than in group II (Figure 8d). In the samples of animals III and V, for which diclofenac 5 mg/kg and CETH 120 mg/kg were used, respectively, the histological picture of the named bones was approaching the norm (Figure 8c,e). At the same time, the highest level of bone mineralization of the medullary region of the tibia was registered in groups III and V of the experimental groups (by Masson trichrome staining).

### 3.8 Immunohistochemistry analysis

In addition to histological analysis using the immunohistochemical method, the level of expression of caspase‐3 was assessed as an indicator of cell death of synovial joint membrane cells.

**FIGURE 9:**
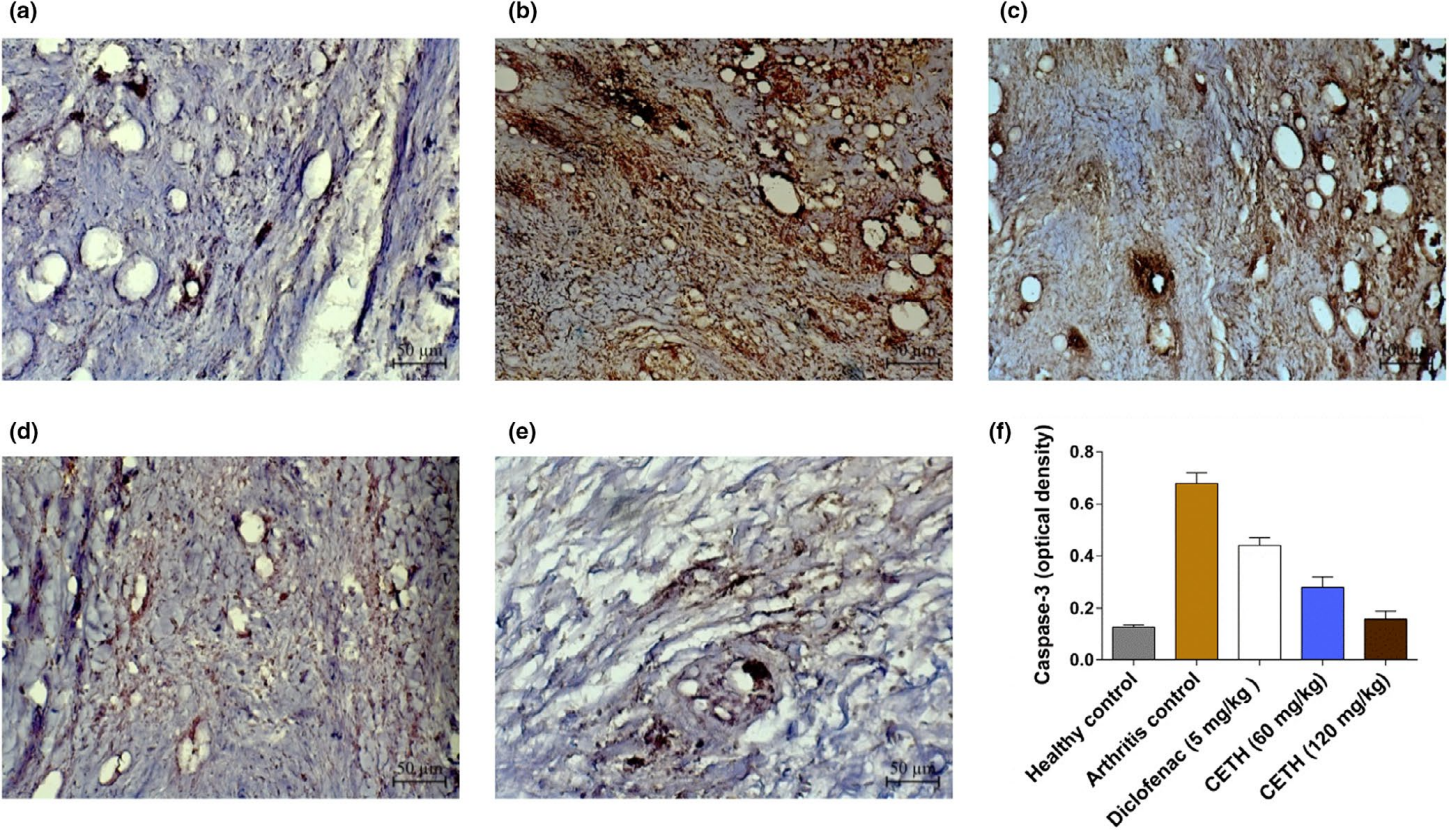
Immunohistochemical expression of caspase‐3 in the synovial membrane of the rat ankle in adjuvant arthritis on day 28, ×200: (a) group I—healthy control. Weak expression of caspase‐3; (b) group II—arthritis control. Super strong expression of caspase‐3; (c) group III—arthritis with diclofenac sodium (5 mg/kg) treatment. Strong expression of caspase‐3; (d) group IV—arthritis with CETH (60 mg/kg) treatment. Moderate expression of caspase‐3; (e) group V—arthritis with CETH (120 mg/kg) treatment. Weak expression of caspase‐3; (f) caspase‐3 expression quantification, expressed as optical density (values represents shown as m ± *SEM*)

In the group of animals of healthy controls, a weak, almost absent staining of the caspase‐3 synovial membrane was observed, which was quantitatively confirmed by the minimum optical density indicator (Figure 9a,f).

Model Group II was characterized by high expression of caspase‐3, as evidenced by intensive brown coloring (Figure 9b). In agreement with the recently obtained results of Shafiey et al. (2018) and Abdel‐Maged et al. (2019), it is logically explained that apoptosis may be one of the mechanisms of progression of rheumatoid arthritis due to the activation of proapoptotic proteins like caspase‐3.

In groups of animals, where diclofenac sodium and CETH were used, on day 28, models of adjuvant arthritis were characterized by less pronounced expression of caspase‐3 (Figure 9c–e). In the order of its reduction and accordance with the results of the average optical density of images, the groups were distributed as follows: group III ˃ group IV ˃ group V (Figure 8f). This pattern of caspase‐3 expression showing the level of apoptosis in periarticular tissues also confirms the decrease in inflammatory alteration in these groups.

## 4 DISCUSSION

Alternatives to control rheumatoid arthritis are becoming increasingly popular (Arnold et al., 2010; Benlidayi et al., 2012; 2018; Grimm et al., 2018; Mobasheri & Turksen, 2019; Wang et al., 2017; Zeuner et al., 2016). With progress in the understanding of the pathophysiology and treatment of rheumatoid arthritis, it has been proven that its nutritional correction is realistic. Dietary approaches can serve as an effective strategy for improving rheumatoid arthritis (Bustamante et al., 2020; Pundarikakshudu, 2019; Vadell et al., 2020).

Of particular interest in this respect are bioactive peptides and peptide‐rich protein hydrolyzates, which represent a new trend in the development of functional foods and nutraceuticals (Chakrabarti et al., 2014; Chalamaiah et al., 2018).

The chicken embryo tissue hydrolyzate (CETH) we are studying has an interesting representation and ratio of oligopeptides and free amino acids with different bioactivity. Therefore, CETH has a promising therapeutic potential for use in pharmacology and nutraceuticals. However, this requires multidirectional experimental validation.

In this study, we have sought to assess the antiarthritic effect of CETH. Hatori et al. (Hatori et al., 2008) studied the antiarthritic effect of casein hydrolyzate without dividing it into components; however, we evaluated the peptide‐amino acid mixture of CETH to assess the complex action of all its components.

On in vitro models, CETH exhibited concentration‐dependent inhibition of protein denaturation, membrane stabilization effect, and inhibitory proteinase activity.

Most researchers report that protein denaturation is one of the reasons for rheumatoid arthritis development due to autoantigen production (Chandra et al., 2015). The expressed property of CETH to block the denaturation of protein even exceeding the NSAID (sodium diclofenac) activity revealed in this study allows us to assert the potential control of the CETH production of autoantigens in rheumatic states.

The stabilizing effect on erythrocyte lysis caused by a hypotonic medium is also a criterion of antiarthritic activity. The erythrocyte membrane is similar to the lysosomal membrane, whose stabilization during the inflammatory process prevents the release of lysosomal enzymes of activated neutrophils, which lead to inflammation progression and tissue damage. One such enzyme of lysosomal granules is proteinases, which enzymatically destroys collagen and proteoglycan matrix of bones and cartilages (Oikonomopoulou et al., 2018). Although the exact mechanisms of membrane protection and inhibition of CETH proteinase are not yet known, it has shown quite a high effect.

The results obtained in vitro prompted us to evaluate the advantages of CETH ex vivo on the animal model for its harmlessness and efficiency.

CETH did not show toxicity in experimental rats, which allowed us to consider it safe for further study on animals.

A model of chronic adjuvant inflammation was used, which is a classic to study the efficacy of antiarthritic drugs. Swelling, inflammatory cell infiltrations, proliferative synovitis, and bone and cartilage structure erosion are clinical signs common to human rheumatoid arthritis and adjuvant‐induced arthritis in rats (Bihani et al., 2014; Noguchi et al., 2008).

Considering that the most reliable methods of assessing the impact of any factors on the body are deservedly considered morphological, and considering that the indicators of the progression of rheumatoid arthritis are radiological and histopathological changes, the antiarthritic effect of CETH has been studied by X‐ray microtomography and histopathological analysis (Grimm et al., 1995).

The study showed that oral administration of CETH to rats weakened arthritis progression and provided effective dose‐dependent correction of morphological changes caused by the adjuvant injection.

Moreover, we compared CETH with the traditional NSAID rheumatoid arthritis treatment with sodium diclofenac. The use of CETH has shown relatively high recovery effects in terms of reduced inflammatory edema, osteolysis inhibition, osteophytosis prevention, periarticular tissue inflammatory response, and cartilage degeneration.

According to the complex of all morphological data obtained by X‐ray micro‐CT and histopathological analysis of the injected leg, the progression of adjuvant arthritis in rats is effectively controlled by CETH treatment (120 mg/kg).

Several mechanisms can logically be assumed to provide the detected effect. In addition to the mechanisms we have registered in vitro, one of the supposed mechanisms of morphologically confirmed antiarthritic effect of CETH may be antioxidant. The role of antioxidants in rheumatoid arthritis therapy has been confirmed by many researchers (Jaswal et al., 2018). The oxidative stress that occurs in an inflamed joint and the decrease in the antioxidant status of the body are the hallmarks of rheumatoid arthritis patients. They contribute to the development of the autoimmune process, and lead to the degradation of connective tissue leading to deformation of the joints and periarticular tissues (Bhowmick et al., 2008).

In addition, active oxygen forms that increase in the late stages of arthritis lead to the induction of synoviocyte and chondrocyte apoptosis (Tak et al., 2000).

Previously, we registered a high antioxidant activity of chicken embryo tissue hydrolyzate, particularly ABTS radical scavenging activity and lipid peroxidation inhibition activity (Rzhepakovsky et al., 2019).

Another suggested mechanism of the CETH antiarthritic effect could be provided by bioactive low molecular weight peptides. CETH contains various di‐ and tripeptides, including the dipeptide proline hydroxyproline (Pro‐Hyp).

According to Lee et al., (2014), pro‐Hyp dipeptides inhibit chondrocyte loss and thinning of articular cartilage caused by the pathology. CETH Hyp can act as a signal for chondrocyte differentiation, thus, providing protection for joint cartilage.

We believe that the antiarthritic effect of CETH does not exclude the role of natural carnosine and anserin dipeptides. According to Drafi et al., (2010), carnosine inhibits the degradation of hyaluronic acid caused by free‐radical processes and corrects the redox imbalance in adjuvant arthritis. In addition, according to Ponist et al., (2016), carnosine may have a systemic antiinflammatory effect in experimental arthritis.

There is evidence that supplements of dipeptide anserin help reduce inflammatory markers in rats with rheumatoid arthritis (Zhao et al., 2020).

According to results, the carnosine and anserin content of CETH is 631.7 ± 16.6 and 24.5 ± 0.6 μg/ml, respectively.

We also believe that the therapeutic efficacy of CETH may be due to the functional peptides of GRKP and QPTIPFFDPQIPK sequences identified by us in its composition, whose immunomodulatory properties have been repeatedly noted in the literature (Cai et al., 2020; Cui et al., 2016).

In addition, the morphologically proven antiarthritic effect of CETH after oral administration can also be provided by its constituent peptides possessing dipeptidyl peptidase IV (DPP IV) inhibitor activity. According to some reports, DPP IV inhibitors can inhibit the progression of rheumatoid arthritis in animal models by inhibiting the proliferation of T‐lymphocytes (Tanaka et al., 1997, 1998). It is believed that DPP IV on activated T cells is a target molecule for rheumatoid arthritis therapy (Williams et al., 2003). However, it is noteworthy that, according to Huang et al., (2012) and to Dudics et al., (2018), some peptides possessing dipeptidyl peptidase IV (DPP IV) inhibitor activity successfully pass through the gastrointestinal tract with the preservation and even increase in this property. According to peptide analysis, CETH includes short‐chain peptides with dipeptidyl peptidase IV inhibitor activity.

The osteoanabolic role of bioactive peptides cannot be ruled out in the detected effect of CETH. According to Amso et al., (2016) and Mada et al., (2018), individual short food peptides can reduce the expression of inflammatory and resorbing cytokines and stimulate differentiation of osteoblasts by increasing the expression of osteogenic genes and the activity of antioxidant enzymes.

Under the conditions of the adjuvant arthritis model used, CETH showed a good osteoanabolic result, which indicates its high potential as an effective strategy in maintaining and correcting bone homeostasis in states characterized by pronounced bone resorption.

Another important factor in ensuring the antiarthritic effect of CETH on chronic arthritis models can be considered as the effect on apoptosis. Apoptosis is regarded as one of the mechanisms involved in the regulation of rheumatoid arthritis. Its role is dual and depends on the stage of arthritis development (Liu, 2003).

The early stages of adjuvant arthritis are characterized by insufficient apoptosis. Starting from the 23rd day, the late stage is characterized by apoptosis activation of synoviocytes and chondrocytes (Bhowmick et al., 2008).

In our study, it would be logical to assume the apoptosis‐inducing activity of CETH, which could be provided by bioactive peptides of embryonic tissue that induce apoptosis processes actively occurring in embryogenesis. An example is the carnosine dipeptide contained in high amounts in CETH, which, according to Pandurangan et al., (2016), has a pronounced apoptosis‐inducing effect due to inhibition of caspase‐3 activity.

However, the current immunohistochemical results for 28 days of the model of adjuvant arthritis in rats indicate overwhelming apoptosis of CETH activity. This may be due to possible nonpeptide inhibitors of caspase‐3 contained in CETH and new bioactive peptides formed as a result of hydrolysis. Besides, the recorded level of apoptosis in periarticular tissues with CETH may be due to a marked decrease in the level of inflammatory alteration, which was recorded by us in the histochemical study. This once again confirms the need for further research to isolate pure compounds from the CETH peptide–amino acid complex and to understand better all the mechanisms involved in its antiarthritic effect.

## 5 CONCLUSIONS

The results obtained at the morphological level confirm the hypothesis that hydrolyzate tissue of the chicken embryo shows a pronounced dose‐dependent antiarthritic effect in adjuvant‐induced joint damage in rats, which is the closest model to rheumatoid arthritis in humans.

This study has a potential theoretical strategy for the safe correction of this pathological process and, for the first time, shows that hydrolyzate of chicken embryo tissue may be a powerful nutraceutical agent or component of a functional food product in the treatment of rheumatoid arthritis.

The therapeutic efficacy of CETH may be due to antioxidant activity, antiinflammatory, osteoanabolic activity, and possible immunotropic action. However, more in‐depth and long‐term studies are needed to finally examine this effect and determine the detailed mechanisms of CETH action at the molecular level.

## CONFLICT OF INTEREST

The authors declare that they have no competing interests.

## AUTHOR CONTRIBUTIONS

**Igor Rzhepakovsky:** Conceptualization (lead); Methodology (lead); Writing‐original draft (equal). **Shahida Siddiqui:** Methodology (equal). **Svetlana Avanesyan:** Conceptualization (equal); Data curation (equal); Methodology (equal). **Mehmet Benlidayi:** Methodology (equal); Validation (equal). **Kunaal Dhingra:** Data curation (equal); Formal analysis (equal). **Alexander Dolgalev:** Project administration (equal); Supervision (equal); Validation (equal). **Natella Enukashvily:** Methodology (equal). **Tilman Fritsch:** Data curation (equal); Methodology (equal). **Volker Heinz:** Writing‐review & editing (equal). **Stanislav Kocherkin:** Conceptualization (equal); Formal analysis (equal); Investigation (equal). **Andrei Nagdalian:** Conceptualization (equal); Supervision (equal). **Marina Sizonenko:** Conceptualization (equal). **Lyudmila Timchenko:** Conceptualization (equal); Investigation (equal); Project administration (equal). **Marco Vukovic:** Methodology (equal). **Sergey Piskov:** Conceptualization (equal); Investigation (equal); Methodology (equal); Project administration (equal); Writing‐original draft (equal). **Wolf Grimm:** Methodology (equal); Project administration (equal); Writing‐original draft (equal).

## ETHICAL STATEMENT

All procedures regarding animal study and design were approved by the Ethics Committee of North Caucasus Federal University and was carried out according to national and university guidelines.
